# The Gut–Iron–Immune Axis in Severe Anaemia and Bacteraemia

**DOI:** 10.3390/nu18172753

**Published:** 2026-08-23

**Authors:** Kelvin Mokaya Abuga, Miranda Y. Bate, Sarah H. Atkinson

**Affiliations:** 1 Kenya Medical Research Institute (KEMRI) Centre for Geographic Medicine Research, KEMRI-Wellcome Trust Research Programme, Kilifi P.O. Box 230-80108, Kenya; kmokaya@kemri-wellcome.org; 2 Sir William Dunn School of Pathology, University of Oxford, Oxford OX1 3RE, UK; miranda.bate@merton.ox.ac.uk; 3 Centre for Tropical Medicine and Global Health, Nuffield Department of Clinical Medicine, University of Oxford, Oxford OX3 7LF, UK; 4 Department of Paediatrics, University of Oxford, Oxford OX3 9DU, UK

**Keywords:** severe anaemia, gut microbiota, bacteraemia, malaria, malnutrition, iron deficiency, environmental enteric dysfunction, iron supplementation, blood transfusion, gut-immune axis

## Abstract

Severe anaemia remains a major public health challenge, disproportionately affecting children and women of reproductive age in sub-Saharan Africa. In high-burden settings, the aetiology of severe anaemia is multifactorial, encompassing nutritional deficiencies, infections, and host genetic factors. Beyond its direct clinical consequences, severe anaemia is also associated with increased susceptibility to invasive bacterial infections, particularly those caused by enteric pathogens such as non-typhoidal *Salmonella* and *Escherichia coli*. In this review, we present an integrated framework linking severe anaemia and invasive bacterial infection through three interconnected biological pathways, collectively termed the gut–iron–immune axis: gut microbial dysbiosis and impaired intestinal barrier integrity; disrupted iron homeostasis; and impaired immune function. We examine context-specific modifiers in endemic settings, including iron deficiency, malnutrition, malaria, sickle cell disease, and environmental enteric dysfunction. We also discuss how management of severe anaemia, including blood transfusion and iron supplementation, reshapes the gut microbiome, with direct implications for microbial translocation, invasive bacterial infection, and clinical outcomes. Finally, we identify key knowledge gaps and research priorities to guide safer and more effective prevention and management of severe anaemia in high-burden settings.

## 1. Introduction

Severe anaemia, characterised by critically low haemoglobin concentrations or red blood cell (RBC) mass [1], remains a major cause of hospitalisation and mortality among young children and women of reproductive age globally [2]. The World Health Organisation defines severe anaemia as a venous haemoglobin concentration <7 g/dL in children aged 6–59 months and pregnant women, and <8 g/dL in individuals aged ≥60 months [1]. Proposed thresholds for neonates are <10.0 g/dL during the first 7 days of life and <8.5 g/dL between 7 and 27 days of age [3]. The burden is disproportionately high in sub-Saharan Africa, where severe anaemia affects up to 14% of community-based children and accounts for 9 to 40% of paediatric hospital admissions with febrile illness [4,5,6]. In this region, severe anaemia is driven by complex and often overlapping aetiologies, including infections, haemoglobinopathies, and nutritional deficiencies [5,6,7]. Beyond its direct clinical consequences, severe anaemia is associated with an increased risk of invasive bacterial infections, particularly those caused by enteric pathogens such as non-typhoidal *Salmonella* (NTS) and *Escherichia coli* [6,7], and findings remain consistent across severe anaemia aetiologies [8,9,10]. Although the biological mechanisms underlying this association remain incompletely understood, accumulating evidence suggests that gut dysfunction, disruption of iron homeostasis, and impaired immunity (collectively the gut–iron–immune axis) may interact to increase susceptibility to invasive bacterial infection [11,12].

**Figure 1:**
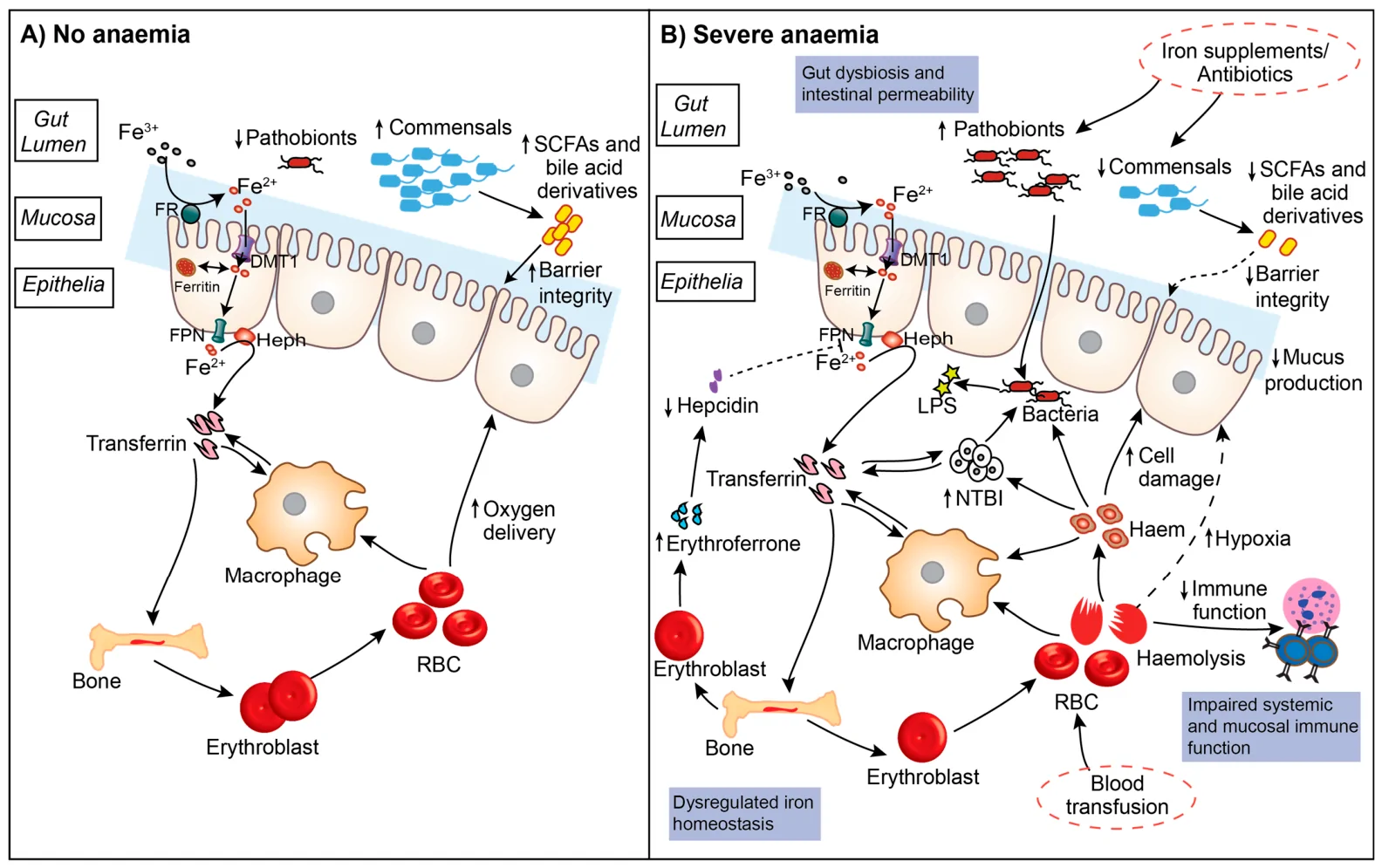
Mechanistic pathways linking severe anaemia to gut dysbiosis and invasive bacterial infection. (**A**) Under physiological conditions, balanced iron homeostasis supports effective erythropoiesis, a diverse short-chain fatty acid (SCFA)-producing gut microbiota, intact epithelial barrier function, and mucosal immune function. Dietary ferric iron is reduced by ferric reductase (FR), transported into enterocytes by divalent metal transporter 1 (DMT1), exported into the circulation by ferroportin (FPN), and oxidised by hephaestin (Heph) for loading onto transferrin. Together, these processes maintain colonisation resistance and limit microbial translocation. (**B**) Severe anaemia promotes gut dysbiosis, compromised barrier integrity, disruption of iron homeostasis, and impaired immune function. These changes may result in expansion of enteric pathobionts, depletion of SCFA-producing commensals, loss of colonisation resistance, increased microbial translocation, and impaired bacterial clearance, culminating in invasive bacterial disease. Therapeutic interventions, including blood transfusion, iron supplementation, and antibiotics, may further modify gut microbial ecology. While individual pathways in this framework are supported by experimental and clinical studies, the combined gut–iron–immune axis remains a proposed conceptual framework that requires validation in longitudinal mechanistic human studies. Solid arrows indicate increased activity; dashed arrows indicate reduced or suppressed activity. Abbreviations: SCFA, short-chain fatty acid; NTBI, non-transferrin-bound iron; RBC, red blood cell; LPS, lipopolysaccharide; FPN, ferroportin; FR, ferric reductase; Heph, hephaestin; DMT1, divalent metal transporter 1.

The gut represents a plausible interface linking severe anaemia with enteric pathogens. Under physiological conditions, the gut microbiome regulates nutrient metabolism, epithelial barrier integrity, mucosal immunity, and colonisation resistance against enteric pathogens [13,14,15]. Emerging evidence suggests that severe anaemia is associated with disruption of the proposed gut–iron–immune axis, encompassing gut dysbiosis, impaired intestinal barrier function, disruption of iron homeostasis, and impaired immunity (Figure 1). Conversely, gut dysbiosis (characterised by reduced microbial diversity, depletion of beneficial commensals, expansion of pathobionts, and altered microbial metabolism) may exacerbate anaemia by impairing iron absorption and utilisation, disrupting micronutrient metabolism, and promoting intestinal and systemic inflammation [13,14]. Microbial translocation and invasive bacterial infection may further exacerbate anaemia through inflammation-driven iron sequestration, haemolysis, bone marrow suppression, and sepsis-mediated eryptosis [16,17,18]. Together, these interactions support a self-reinforcing cycle linking severe anaemia, gut dysfunction, and invasive bacterial infection. In high-burden settings, this cycle is likely to be modified by contextual factors including undernutrition, malaria, environmental enteric dysfunction (EED), and sickle cell disease, which influence both anaemia susceptibility and the gut microbiome [19,20,21].

In this Review, we propose that disruption of the gut–iron–immune axis represents a unifying biological framework linking severe anaemia to invasive bacterial infection. We synthesise current evidence on the bidirectional biological mechanisms linking severe anaemia, iron homeostasis, and the gut microbiome; examine how these interactions contribute to susceptibility to invasive bacterial disease; and discuss priorities for future mechanistic and translational research.

## 2. The Gut Microbiome in Health

The gut microbiome is a complex and spatially heterogeneous intestinal ecosystem comprising bacteria, archaea, viruses, fungi and protozoa [14]. Its microbial component, the gut microbiota, consists of approximately 10^13^–10^14^ microbial cells in the adult human gut, with the highest microbial densities in the colon [22,23]. The gut microbiota has co-evolved with the host to form a dynamic mutualistic ecosystem [24,25]. Although microbial composition varies considerably between individuals and over time, bacterial communities typically span approximately 50 phyla and are dominated by Bacillota (formerly Firmicutes) and Bacteroidota (formerly Bacteroidetes) [14,19].

Functionally, the gut microbiota regulates host nutrient metabolism, iron homeostasis, immune function, and intestinal barrier integrity [13,15,20]. One of the principal metabolic functions of the gut microbiota is the fermentation of dietary fibre into short-chain fatty acids (SCFAs), principally acetate, propionate, and butyrate [26]. These metabolites support epithelial energy metabolism, modulate mucosal and systemic immune responses, and strengthen barrier integrity [27]. The microbiota also converts primary bile acids, amino acids, and dietary phytochemicals into bioactive metabolites that regulate epithelial regeneration, antimicrobial peptide production, and mucosal immune tolerance [28,29]. In addition, the gut microbiota synthesises vitamin K and several B vitamins [30]. Vitamin B12 and folate are essential cofactors for DNA synthesis and erythropoiesis [31,32]. Although microbially synthesised folate may contribute modestly to host folate status [33], most microbial vitamin B12 is synthesised in the colon, distal to the primary site of vitamin B12 absorption in the terminal ileum [34]. Consequently, host vitamin B12 status remains largely dependent on dietary intake.

The gut microbiota is also an important regulator of iron homeostasis: it influences luminal iron availability, intestinal iron absorption, and systemic iron-regulatory pathways [13]. Within the intestinal lumen, iron availability shapes microbial ecology and host–pathogen interactions. Many enteric bacteria, particularly members of Enterobacteriaceae, produce high-affinity siderophores that scavenge ferric iron from the intestinal microenvironment [35,36], providing a competitive advantage under iron-limited conditions [37]. Experimental and clinical studies show that microbial metabolism products, such as SCFAs, increase iron solubility by lowering luminal pH, potentially enhancing intestinal iron absorption [38,39]. Beyond the intestine, experimental studies suggest that the gut microbiota contributes to systemic iron homeostasis by modulating bone marrow iron uptake and hepcidin-mediated control of intestinal iron absorption and iron recycling [27,40]. Experimental studies further suggest that the gut microbiota promotes the local dendritic cell production of hepcidin [41] and hepcidin-inducing signals [42]. Conversely, the host limits microbial access to iron through nutritional immunity, whereby proteins such as hepcidin, transferrin, lactoferrin, and lipocalin-2 restrict iron availability while maintaining systemic iron homeostasis [43,44].

These metabolic activities are closely integrated with maintenance of intestinal barrier integrity and mucosal immunity. The intestinal barrier comprises a single layer of epithelial cells reinforced by tight junctions, a mucus layer, antimicrobial peptides, secretory immunoglobulin A (IgA), and specialised resident immune cells [20,45,46]. Continuous crosstalk between the microbiota, epithelial cells, and resident immune cells coordinates intestinal homeostasis and immune surveillance, tolerance, and protection [20,47]. Through interactions with the host’s pattern-recognition receptors, microbial-associated molecular patterns of the gut microbiota support the development, maturation, and function of both mucosal and systemic immune cells [15,20,46].

**Figure 2:**
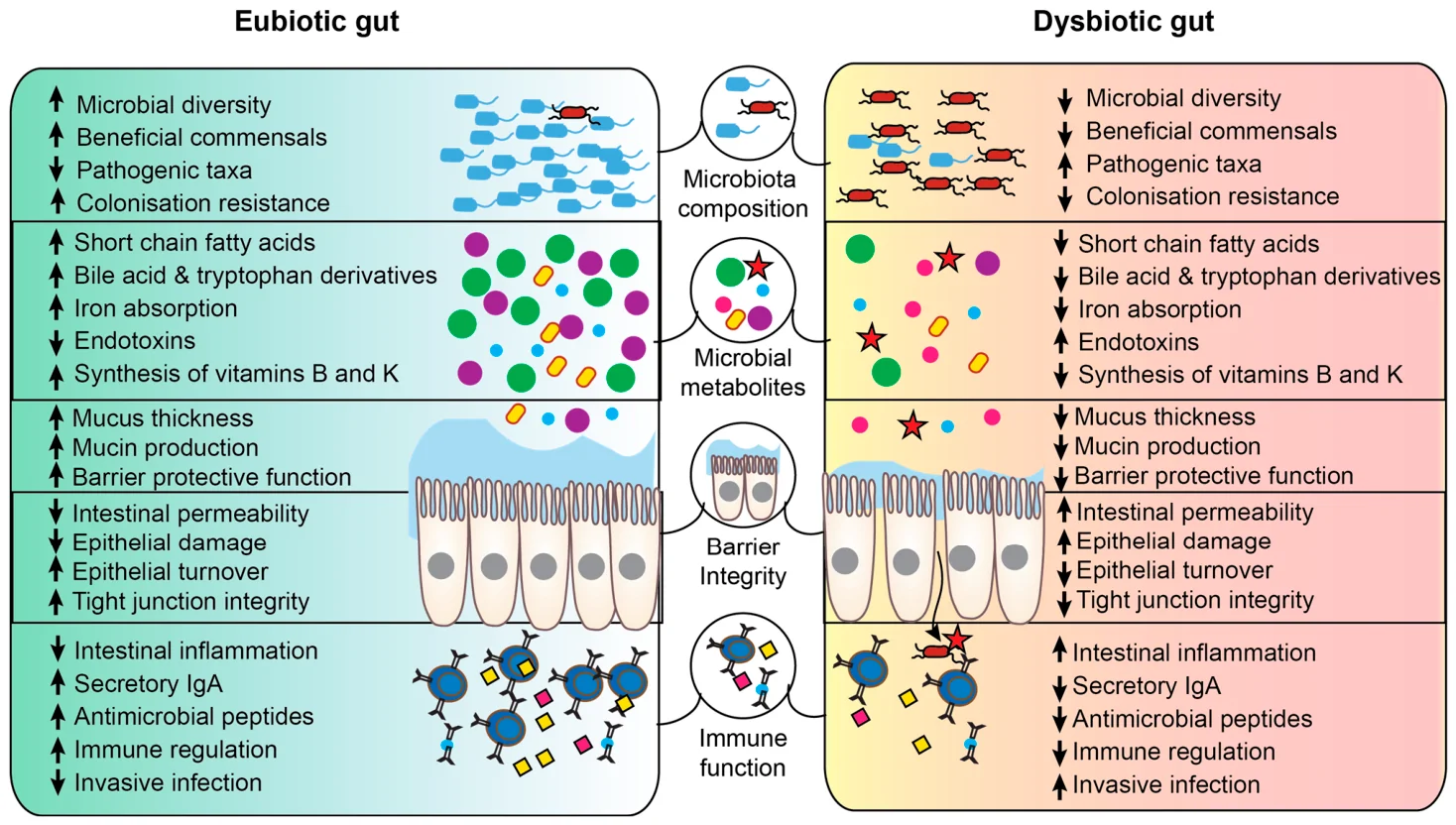
Role of the gut microbiome in severe anaemia and invasive bacterial infection. (**Left panel**): A healthy gut microbiome (eubiotic gut) supports iron homeostasis, nutrient metabolism, epithelial barrier integrity, and mucosal immunity, thereby maintaining colonisation resistance and limiting microbial translocation. (**Right panel**): Gut dysbiosis disrupts these processes through impaired iron absorption and metabolism, reduced short-chain fatty acid production, compromised intestinal barrier integrity, and dysregulated immune responses, contributing to severe anaemia and increasing susceptibility to invasive bacterial infection. Abbreviations: IgA, immunoglobulin A. Upwards arrows (↑) indicate increased activity; downward arrows (↓) indicate reduced or suppressed activity.

Together, the metabolic, barrier, and immune functions of a healthy microbiota maintain colonisation resistance. Resident commensals suppress expansion of enteric pathogens through competition for nutrients and ecological niches, production of antimicrobial metabolites, maintenance of luminal oxygen and pH, and sequestration of essential micronutrients, particularly iron [48]. Loss of colonisation resistance promotes expansion of enteric pathobionts, increasing the risk of microbial translocation and invasive bacterial infection (Figure 2).

## 3. Severe Anaemia and the Gut Microbiota

Severe anaemia and the gut microbiota are linked through complex bidirectional interactions. Current evidence suggests that gut dysbiosis may increase risk of anaemia, while severe anaemia may also alter gut microbial composition and function. The following sections examine both directions of this relationship before considering disease-specific mechanisms.

### 3.1. Gut Dysbiosis as a Contributor to Anaemia

Gut dysbiosis may contribute to anaemia through impaired intestinal iron absorption, reduced production of microbial metabolites that support epithelial integrity and nutrient metabolism, chronic intestinal inflammation, hepcidin-mediated iron sequestration, and ineffective erythropoiesis (Figure 2). These mechanisms have been comprehensively reviewed elsewhere [13,49,50,51]. In experimental studies, disruption of the gut microbiota, including through broad-spectrum antibiotic exposure, impairs haematopoiesis and exacerbates anaemia [52]. However, direct evidence in humans remains sparse and is largely derived from a small number of observational studies in pregnancy, chronic kidney disease, or inflammatory bowel disease [53,54,55,56]. Mendelian randomisation analyses, conducted predominantly in populations of European ancestry, have provided preliminary evidence supporting a potentially causal relationship between specific microbial taxa, including *Ruminococcus*, *Desulfovibrio*, and *Roseburia*, and increased susceptibility to iron deficiency anaemia (IDA) [57,58,59,60]. However, these findings may not be generalisable to populations in sub-Saharan Africa where gut microbial composition, diet, and infectious disease burden differ substantially. Thus, whether the gut microbiota similarly contributes to severe anaemia pathogenesis in African children remains uncertain.

### 3.2. Severe Anaemia as a Driver of Gut Dysbiosis

Accumulating evidence indicates that severe anaemia is associated with remodelling of the gut microbiome and intestinal barrier integrity. In experimental studies, severe anaemia compromises intestinal barrier integrity through hypoxia-induced epithelial injury, intestinal inflammation, and reduced expression of tight-junction proteins, including zonula occludens-1 [61,62]. These changes are likely to alter the intestinal microenvironment, promoting microbial dysbiosis and weakening colonisation resistance. Although evidence in humans remains limited, studies in young children and preterm infants report a dysbiotic microbial profile in anaemia, characterised by reduced microbial diversity, depletion of beneficial commensals, and expansion of enteric pathobionts [63,64,65,66]. The extent of these alterations appears to increase with anaemia severity. For example, among preterm infants in the United States, increasing severity of anaemia was associated with progressive depletion of Bacillota and enrichment of Pseudomonadota [66].

Beyond taxonomic changes, severe anaemia may also be associated with functional alterations in the intestinal ecosystem, including increased bacterial virulence potential and intestinal injury [67,68,69]. However, evidence remains sparse, and existing studies are observational and conducted predominantly in preterm infants. It also remains unclear whether these intestinal alterations are drivers or consequences of severe anaemia or reflect underlying disease factors. Furthermore, current evidence is derived predominantly from 16S rRNA gene sequencing, which characterises microbial taxonomic composition but provides limited insight into microbial function. Consequently, it remains uncertain whether taxonomic alterations translate into biologically meaningful changes relevant to disease pathogenesis. Complementary multi-omic approaches, including shotgun metagenomics, metatranscriptomics, and metabolomics [70], are therefore needed to define the functional consequences of gut dysbiosis.

Although severe anaemia is a shared clinical phenotype, its underlying causes differ substantially in their effects on iron metabolism, haemolysis, inflammation, and immune function [11]. Despite these differences, broadly similar microbial signatures have been reported across multiple anaemia phenotypes, including iron deficiency anaemia (IDA) [71,72,73,74,75], sickle cell anaemia (SCA) [76,77,78,79,80], severe malarial anaemia (SMA) [81], anaemia of chronic disease [82,83], and aplastic anaemia [84,85]. Whether these shared microbial alterations are attributable to severe anaemia itself or reflect disease-specific pathophysiological mechanisms remains uncertain. The following sections examine how major causes of severe anaemia in children living in sub-Saharan Africa, including iron deficiency, SMA, and SCA (Table 1), may lead to gut dysbiosis.

### 3.3. Gut Microbiome Alterations Across Major Aetiologies of Severe Anaemia

**Iron deficiency** is the most common cause of anaemia globally [3,86]. Observational studies have linked IDA with the common dysbiotic microbial signature described above, characterised by reduced microbial diversity, depletion of beneficial commensals, increased intestinal inflammation, disturbances in microbial metabolism, and expansion of pathobionts [71,72,73,74,75]. However, most existing evidence is derived from cross-sectional studies. Mendelian randomisation studies, predominantly in European ancestry populations, report inconsistent findings. Some studies found associations between genetically predicted iron status and gut taxa (including Ruminococcaceae), while others show little or no evidence for causal effects [57,58]. In contrast, experimental studies consistently support adverse effects of IDA on the gut microbiota. In animal models, IDA remodels gut microbial composition, promotes small intestine bacterial overgrowth, alters SCFA production, and disrupts epithelial barrier integrity [87,88,89,90,91]. In vitro studies further demonstrate that iron limitation suppresses microbial pathways involved in SCFA biosynthesis [92]. These alterations appear partially reversible following nutritional interventions in experimental models, including iron supplementation and dietary modification, such as fermented goat’s milk [93,94].

Mechanistically, the relationship between iron availability and the gut microbiome appears to be U-shaped [95]. Iron deficiency may promote dysbiosis through reduced luminal iron availability and its downstream effects on microbial ecology and host physiology [13]. Conversely, oral iron supplementation may increase the amount of unabsorbed iron within the intestinal lumen, providing a growth advantage for siderophilic pathobionts [96,97]. These observations suggest that iron availability is a major ecological determinant of gut microbial composition and function, providing a plausible link between IDA, gut dysbiosis, and susceptibility to invasive bacterial infection.

**Severe malarial anaemia** (SMA) remains a leading cause of hospitalisation and death, particularly among young African children [98]. SMA arises from *Plasmodium*-driven haemolysis of parasitised and non-parasitised erythrocytes, bone marrow suppression, and inflammation-mediated dyserythropoiesis [99,100]. Evidence linking SMA to the gut microbiome remains limited. In Ugandan children with SMA, stool microbiota demonstrated enrichment of enteric pathobionts, including *E. coli* and *Klebsiella pneumoniae* [81]. SMA has also been associated with increased intestinal permeability and endotoxaemia in Kenyan children [101], findings that are consistent with impaired intestinal barrier function and microbial translocation. These alterations may contribute to the well-recognised susceptibility of children with SMA to invasive NTS bacteraemia [8,102,103]. However, increased dysbiosis and intestinal permeability has also been reported in uncomplicated and severe malaria regardless of severe anaemia [104,105,106], suggesting that malaria itself contributes to gut barrier dysfunction. The relative contributions of severe anaemia, malaria parasite pathogenesis, or haemolysis to intestinal dysbiosis remain uncertain.

**Sickle cell anaemia** (SCA) is a hereditary haemolytic disorder characterised by chronic anaemia, recurrent vaso-occlusion, dysregulated iron metabolism, and persistent inflammation [107]. Although human data remains sparse, SCA is consistently associated with gut dysbiosis characterised by reduced microbial diversity, depletion of SCFA-producing commensals, enrichment of pathobionts including *Clostridium* XI, *Prevotella* spp., and Enterobacteriaceae, and impaired intestinal barrier function [76,77,78,79,80]. Experimental studies support these SCA-mediated gut effects [79,108,109]. These microbial alterations are thought to arise from chronic intestinal hypoxia, repeated ischaemia–reperfusion injury caused by vaso-occlusion, haem exposure, altered iron handling, and sustained inflammatory stress [110]. The resultant gut dysbiosis amplifies systemic inflammation, vaso-occlusion, and pain in SCA patients [110,111,112]. In murine models, iron-restricted dietary interventions improve gut barrier integrity and partially reverse SCA-mediated dysbiosis [113]. Disease-modifying therapies, including hydroxyurea, are also associated with partial restoration of beneficial microbial taxa and improved intestinal homeostasis [114].

**Malnutrition** is a major determinant of severe anaemia in low-resource settings, and lower haemoglobin levels in malnourished children are strongly associated with increased risk of invasive bacterial infections [6]. Depending on nutritional phenotype, severe anaemia results from deficiencies in key substrates for erythropoiesis (including iron, folate, vitamin B12, and protein), inflammation-mediated iron sequestration, oxidative stress–mediated haemolysis, or impaired bone marrow responsiveness [115,116,117]. Direct evidence linking malnutrition-associated anaemia to gut microbial disruption is sparse, although malnutrition is consistently associated with microbial immaturity and gut dysbiosis [118,119,120]. These alterations provide biological plausibility that malnutrition may modify the relationship between severe anaemia, the gut microbiome, and susceptibility to invasive bacterial infection. In low-resource settings, both anaemia and malnutrition are also linked with EED (also termed environmental enteropathy) [121,122,123], a chronic subclinical disorder of the small intestine characterised by villous blunting, epithelial dysfunction, and reduced barrier capacity [124]. EED may further increase intestinal permeability, facilitate microbial translocation, and amplify susceptibility to invasive bacterial infection [19].

### 3.4. Summary of Existing Evidence

Taken together, current evidence indicates that severe anaemia, irrespective of its underlying aetiology, is associated with similar alterations in the gut microbiome. However, the underlying mechanisms of this gut dysbiosis differ by aetiology: IDA primarily alters systemic and luminal iron availability; SMA is characterised by haemolysis and parasite-induced inflammation; SCA by vaso-occlusion and chronic haemolysis; and malnutrition by nutrient deficiency and oxidative stress-mediated haemolysis (Table 2). Despite this, the collective evidence suggests convergence on a limited number of downstream processes, including altered microbial ecology, impaired barrier integrity, disrupted iron homeostasis, and intestinal inflammation. This convergence raises the possibility that severe anaemia contributes to a shared intestinal phenotype, although the relative contributions of severe anaemia itself and disease-specific mechanisms remain uncertain. Current evidence is largely associative. Differences in anaemia definitions, underlying disease aetiologies, study populations, sequencing platforms, targeted 16S rRNA hypervariable regions, and bioinformatic pipelines further limit direct comparison between studies (Table 1). Longitudinal mechanistic studies integrating microbiome, immune, and iron homeostasis data are needed to determine whether these shared intestinal alterations contribute to the increased susceptibility to invasive bacterial infection observed in severe anaemia.

## 4. Mechanisms Linking Severe Anaemia to Gut Dysbiosis and Invasive Infection

Several interconnected mechanisms likely underpin the development of gut dysbiosis and increased susceptibility to invasive infection in severe anaemia (Figure 1). Central to this framework are intestinal tissue hypoxia, disrupted iron and nutrient homeostasis, compromised epithelial barrier integrity, and impaired mucosal and systemic immune responses (Box 1).

Box 1 **Potential consequences of severe anaemia on the gut microbiome, intestinal barrier, and host immunity.**

Effects on the gut microbiota

- Reduced microbial diversity;
- Reduced abundance of commensal and beneficial taxa;
- Expansion of facultative anaerobes and enteric pathobionts;
- Altered microbial metabolic capacity;
- Reduced production of SCFAs, bile acid, indole, and amino acid-derived metabolites;
- Impaired colonisation resistance against enteric pathogens;
- Altered microbial iron acquisition and siderophore-mediated competition.

Effects on mucosa and epithelia

- Reduced oxygen delivery to the intestinal mucosa;
- Reduced mucus production and mucus layer integrity;
- Increased epithelial cell damage;
- Impaired intestinal barrier integrity and increased permeability;
- Impaired epithelial cell turnover and repair;
- Increased intestinal inflammation;
- Reduced mucosal immune function, including secretory IgA and antimicrobial peptide production;
- Increased microbial translocation and endotoxaemia.

Systemic effects

- Increased susceptibility to invasive bacterial infection;
- Impaired innate and adaptive immune cell function;
- Increased systemic inflammation;
- Dysregulated iron homeostasis and hepcidin signalling;
- Increased NTBI and free haem availability for invasive pathogens;
- Altered gut–immune signalling.

Reduced haemoglobin concentrations impair tissue oxygen delivery, including to the gastrointestinal tract. Intestinal hypoxia activates hypoxia-inducible factor signalling, an adaptive response that promotes mucus secretion, antimicrobial peptide production, epithelial metabolism, and tight-junction integrity [125,126]. However, persistent or severe hypoxia may overwhelm these protective mechanisms, ultimately compromising the intestinal barrier [126]. In addition, reduced epithelial oxygen consumption, compounded by depletion of butyrate-producing commensals, increases oxygen diffusion into the intestinal lumen [127,128]. This “oxygen leak” favours facultative anaerobes, particularly Enterobacteriaceae, at the expense of obligate anaerobic commensals, thereby promoting gut dysbiosis [128,129]. In turn, gut dysbiosis may further impair intestinal barrier function, potentially reinforcing the cycle linking severe anaemia and intestinal dysfunction.

Disruption of iron homeostasis amplifies intestinal dysfunction through multiple interconnected mechanisms. Iron is a fundamental determinant of microbial competition [35]. Altered iron status, whether arising from iron deficiency, iron sequestration, haemolysis, or iron supplementation, may reshape the intestinal microbiota composition [95]. For example, NTBI and free haem released during haemolysis provide a readily accessible nutrient source for invasive pathogens [130,131], whereas changes in luminal iron availability influence competition between commensals and pathobionts. Enterobacteriaceae are particularly well adapted to these changes because they possess highly efficient iron-acquisition systems, including multiple siderophores and haem uptake pathways [36]. In parallel, accelerated erythropoiesis suppresses production of the iron-regulatory hormone hepcidin [102,132,133], altering intestinal iron absorption and systemic iron recycling, with secondary effects on luminal iron availability and microbial ecology [51]. The resulting gut dysbiosis may further disrupt iron homeostasis by impairing intestinal iron absorption, altering microbial iron metabolism, promoting chronic intestinal inflammation, and enhancing hepcidin-mediated iron sequestration [13,95].

Progression to bacteraemia requires microbial translocation across the intestinal epithelium. Under physiological conditions, the intestinal barrier restricts intestinal microorganisms from accessing the systemic circulation. In severe anaemia, multiple interacting processes may compromise this barrier. NTBI and free haem can promote oxidative epithelial injury through generation of reactive oxygen species [134,135]. Experimental models also show that severe anaemia reduces expression of epithelial tight-junction proteins such as zonula occludens-1 [61,62]. Concurrent gut dysbiosis, characterised by reduced production of SCFAs and other microbiota-derived metabolites, may further impair epithelial integrity and mucosal defence. Expansion of enteric pathobionts, driven by altered oxygen and iron availability, increases bacterial adherence, epithelial invasion, and inflammatory injury (Figure 1). Together, these interacting mechanisms may disrupt intestinal barrier function and facilitate microbial translocation.

Establishment of invasive bacterial infection ultimately depends on host immune competence. Under physiological conditions, translocation of small numbers of intestinal microorganisms is rapidly contained by coordinated mucosal and systemic immune responses [15,46]. Severe anaemia compromises these immune mechanisms at multiple levels. Reduced barrier protection, as discussed above, diminishes containment of enteric pathogens at the epithelial surface. Severe anaemia is also associated with impaired innate immune function, including macrophage activation, cytokine signalling, and neutrophil chemotaxis, phagocytosis, and oxidative burst, which reduces bacterial killing [11,12,136].

Evidence regarding adaptive immune dysfunction in severe anaemia remains comparatively limited, but available studies suggest impaired B- and T-cell development and function [11,135]. Low haemoglobin concentrations have been associated with reduced T-cell frequencies and impaired humoral bactericidal activity in children [137,138]; severe iron deficiency has been linked to reduced antigen-specific antibody responses, diminished mature B-cell populations, thymic atrophy, and impaired T-cell function [139,140,141], while severe malarial anaemia is associated with elevated concentrations of pro-inflammatory cytokines [142,143,144]. Experimental studies further demonstrate that iron deficiency within intestinal regulatory T cells disrupts immune homeostasis and can precipitate lethal autoimmune disease [145]. In disease-specific contexts, functional asplenia in SCA further impairs clearance of invasive bacteria [107,146]. Finally, gut dysbiosis and microbial translocation may amplify immune dysregulation through persistent inflammatory signalling and altered microbial metabolite production [15,20,46]. Taken together, these reciprocal interactions may increase the likelihood that translocating enteric pathogens survive, disseminate, and establish bloodstream infection in individuals with severe anaemia.

Validation of the proposed gut–iron–immune axis will require integration of biomarkers that capture intestinal barrier integrity, microbial translocation, microbial metabolism, iron homeostasis, and host immune responses (Box 2).

Box 2 **Candidate biomarkers to evaluate the gut–iron–immune axis in severe anaemia.**

Biomarkers of intestinal epithelial injury, barrier dysfunction, and inflammation [147,148]

- Intestinal fatty acid-binding protein (I-FABP);
- Zonulin;
- Faecal calprotectin;
- Claudin and occludin fragments;
- Lactulose:mannitol ratio (or lactulose:rhamnose ratio);
- Regenerating islet-derived protein 3α (REG3α).

Biomarkers of microbial translocation [147,148]

- Plasma lipopolysaccharide (LPS);
- Lipopolysaccharide-binding protein (LBP);
- Endotoxin core antibodies (EndoCAb);
- Bacterial DNA;
- Circulating 16S rRNA gene copies.

Biomarkers of microbial metabolism [147,148]

- Faecal short-chain fatty acids;
- Secondary bile acid profiles;
- Tryptophan and other microbial metabolites.

Biomarkers of iron homeostasis [149,150,151]

- Ferritin;
- Transferrin and transferrin saturation;
- Hepcidin;
- Erythroferrone;
- Soluble transferrin receptor;
- Serum iron;
- Non-transferrin-bound iron;
- Total iron binding capacity.

Biomarkers of immune dysfunction [152]

- Leukocyte immunophenotyping;
- Markers of monocyte and macrophage activation (e.g., soluble CD14 and soluble CD163);
- Neutrophil function assays;
- Inflammatory cytokines and chemokines.

## 5. Therapeutic Interventions in Severe Anaemia

Current management of severe anaemia focuses on rapidly restoring haemoglobin concentrations through blood transfusion followed by iron supplementation while addressing the underlying causes of anaemia [153,154]. Although these interventions are often lifesaving, emerging evidence indicates that they also modify the gut microbiome, with potential consequences for gut microbial ecology, intestinal barrier function, and susceptibility to infection. Treatment of the underlying causes of severe anaemia may further alter the gut microbiome [114,155], although evidence in the context of severe anaemia remains limited. Understanding these microbiome-mediated effects could facilitate the development of therapeutic strategies that optimise haematological recovery while minimising unintended adverse effects on the gut.

### 5.1. Blood Transfusion

Blood transfusion rapidly restores oxygen-carrying capacity and remains the primary treatment for life-threatening severe anaemia. Unlike oral treatments, transfusion bypasses the intestinal lumen and, therefore, avoids directly disrupting the gut microbiota. Nevertheless, indirect effects on gut microbial ecology are biologically plausible.

Although evidence is limited, transfusion may positively influence the gut microbiome. In patients with traumatic haemorrhage, blood transfusion has been associated with increased gut microbial α- and β-diversity [156]. However, interpretation of these findings is complicated by the frequent co-administration of empiric antibiotics and other intensive care interventions in these patients, which independently exert more immediate effects on the gut microbiota [157]. Experimental studies provide stronger mechanistic support. In murine haemorrhage models, whole-blood transfusion partially reversed intestinal dysbiosis, likely by reversing anaemia-associated hypoxia [158].

Transfusion could also, theoretically, have unintended adverse effects on the intestinal environment. Breakdown of transfused RBCs can transiently increase circulating NTBI and free haem. These molecules may promote epithelial injury and provide an accessible iron source for enteric pathobionts (Figure 1). Supporting this, blood transfusion has been associated with intestinal injury and necrotising enterocolitis in preterm neonates [159]. However, it remains uncertain whether these complications are caused by blood transfusion itself or the underlying severe anaemia and tissue hypoxia [160]. Prospective longitudinal studies are needed to define how blood transfusion interacts with the gut microbiome and whether these effects influence infection risk, recovery, or survival.

### 5.2. Iron Supplementation

Oral iron supplementation, alone or in combination with folic acid, remains the cornerstone of anaemia treatment [154]. Home-based food fortification with iron, either alone or as multiple micronutrient powders, consistently improves iron status and reduces anaemia in young children [161]. Accordingly, the World Health Organization recommends routine iron supplementation in populations with a high prevalence of anaemia [162]. However, not all orally administered iron is absorbed. Unabsorbed iron passes into the distal intestine, where it serves as a potent ecological substrate that influences microbial growth, composition, and interspecies competition [51].

Luminal iron levels can directly reshape the gut microbiota composition by altering the competitive landscape between commensals and pathobionts [13]. In several studies conducted in low-resource settings, iron supplementation or food fortification promoted expansion of pathogenic Enterobacteriaceae, depletion of *Bifidobacterium* and other SCFA-producing commensals, increased intestinal inflammation, and a higher incidence of diarrhoeal disease [96,97,163,164,165] (Table 3). Similar observations have been reported elsewhere. For example, Swedish infants receiving high-iron formula exhibited reduced bifidobacterial abundance [166]. However, interpretation of these findings is limited because most studies were relatively short in duration, not conducted specifically in patients with severe anaemia, used heterogeneous iron formulations and doses, and relied predominantly on 16S rRNA gene sequencing (Table 3).

The adverse effects of iron supplementation have not been universally observed. For example, studies in children from Bangladesh and South Africa [167,168], as well as adults from high-income settings [169,170,171], reported little or no effect of iron supplementation on microbial composition, faecal SCFA, or gut inflammation. Similarly, experimental animal iron supplementation studies have produced heterogeneous findings, ranging from pronounced dysbiosis [172,173,174], to minimal or no observable microbiome alterations [91,175]. Such variability likely reflects differences in baseline iron status, iron formulation and dose, age, dietary context, pre-existing microbiome composition, and other potential confounding factors.

Iron-induced microbiome disruption is most relevant in populations already at highest risk of severe anaemia, infection, and EED. This presents a critical therapeutic challenge: how can iron deficiency and anaemia be corrected while preserving a healthy gut microbiome? Several alternative strategies show promise. Co-administration of ferrous sulphate with prebiotic oligosaccharides had minimal effects on gut microbial composition in Kenyan children [165]. In experimental models, intravenous iron sucrose produced fewer microbiome perturbations than oral iron sulphate in adults with inflammatory bowel disease [176], while ferric maltol caused less dysbiosis than ferrous sulphate [177]. Other emerging approaches, including intravenous iron supplementation, precision iron formulations, and lower-dose regimens warrant further evaluation.

### 5.3. Microbiome-Directed Interventions

Microbiome-directed interventions include prebiotics, probiotics, synbiotics, postbiotics, faecal microbiota transplantation, and dietary modification [178,179,180]. However, evidence supporting these approaches in anaemia remains limited and inconsistent. Systematic reviews of prebiotics, probiotics, synbiotics, and postbiotics have reported conflicting findings, with some demonstrating modest improvements in haemoglobin levels [180] and others reporting no significant clinical benefit [181,182]. Faecal microbiota transplantation has shown preliminary benefit in restoring microbial diversity and improving haemoglobin concentrations in adults with anaemia of chronic disease [82]. Dietary strategies designed to modulate the gut microbiome, including fermented goat’s milk and iron-fortified cereals, have shown promise in experimental studies [93] but evidence in humans remains limited and conflicting [183,184]. Taken together, the therapeutic potential of microbiome-directed strategies remains promising but unproven, and adequately powered mechanistic clinical trials are required to establish their efficacy, safety, and optimal role in the management of severe anaemia.

## 6. Conclusions and Recommendations

### 6.1. Strengths and Limitations of Current Evidence

Despite growing recognition of the gut microbiome as a key regulator of host metabolism and immunity, its role in the pathophysiology of severe anaemia remains incompletely understood. Current evidence suggests that severe anaemia and gut dysbiosis are linked through a complex interplay between disrupted iron homeostasis, impaired intestinal barrier function and altered immune responses. However, several important limitations of the current evidence should be considered when interpreting this proposed framework. The available evidence base is limited by predominantly cross-sectional study designs, relatively small sample sizes, and residual confounding, precluding robust inference regarding temporality and causality. Heterogeneity in study populations, geographical settings, anaemia definitions, dietary patterns, antibiotic exposure, sequencing platforms, bioinformatic pipelines, and analytical methods further limits direct comparison across studies. Most studies relied on 16S rRNA gene sequencing, which characterises microbial taxonomic composition but provides limited insight into microbial function or host–microbe interactions. Evidence regarding the gut mycobiome, virome, and bacteriophages in severe anaemia remains scarce, despite increasing recognition of their roles in intestinal barrier integrity, microbial ecology, host immunity, and colonisation resistance [185,186,187]. Few studies have integrated microbiome profiling with host immune phenotyping or complementary multi-omic approaches, including metagenomics, metatranscriptomics, metabolomics, and metaproteomics. Finally, evidence on direct associations between severe anaemia and the gut microbiome remain extremely limited, with much of the available evidence extrapolated from underlying aetiologies, such as IDA, malaria, SCA, and malnutrition. Each of these conditions has distinct pathophysiological mechanisms that may independently influence the gut microbiome.

### 6.2. Priorities for Future Research

Addressing these knowledge gaps requires a shift towards longitudinal, integrative, and mechanistically informed studies, particularly in populations with the highest burden of severe anaemia. Such work must move beyond taxonomic profiling to incorporate serial assessments before and after treatment, including blood transfusion, iron supplementation, antimicrobial exposure, and nutritional rehabilitation (Box 3). Equally important is the systematic measurement of mucosal and systemic immune responses to map the immune circuitry linking anaemia to gut dysbiosis. Integrated multi-omics approaches combining metagenomics, metabolomics, transcriptomics, proteomics, and host phenotyping will be essential to define functional host–microbe interactions that compositional profiling alone cannot capture.

A better understanding of the gut–iron–immune axis in severe anaemia may inform the development of diagnostic biomarkers to identify patients at increased risk of invasive bacterial infection, guide personalised iron supplementation strategies that minimise adverse effects on the gut microbiome, and support the development of microbiome-directed adjunctive therapies. Although these applications remain investigational, they have the potential to improve risk stratification, inform targeted management of high-risk patients, and reduce morbidity and mortality associated with severe anaemia in high-burden settings.

Box 3 **Key gaps in the gut–iron–immune axis.**

Causality and disease mechanisms

- What are the temporal relationships between severe anaemia, gut dysbiosis, intestinal barrier dysfunction, and invasive infection?
- Are alterations of the gut microbiome reversible following correction of severe anaemia?
- Which microbial functions and host–microbe interactions drive susceptibility to invasive bacterial infection?
- What roles do the gut virome, mycobiome, and bacteriophages play in severe anaemia and susceptibility to invasive bacterial infection?
- Iron homeostasis and microbial ecology
- How do changes in luminal and systemic iron availability influence microbial community structure, function, and pathogen colonisation?
- What are the relative contributions of iron deficiency, hepcidin-mediated iron sequestration, haemolysis, and dietary iron exposure to gut dysbiosis?
- Which microbial functional pathways are most sensitive to disruptions in iron metabolism?

Context-specific determinants in high-burden settings

- How do microbial metabolic functions compare across major severe anaemia aetiologies, including iron deficiency, malaria-associated anaemia, and malnutrition?
- How does EED modify severe anaemia–gut microbiome interactions?
- Are the mechanisms linking severe anaemia and gut dysbiosis consistent across different aetiologies, epidemiological settings, and age groups?

Microbiome informed therapies and biomarker development

- How do iron supplementation, blood transfusion, and emerging iron-modulating therapies influence gut microbial ecology, intestinal barrier function, and infection risk?
- Can microbiome-directed interventions (prebiotics, probiotics, synbiotics, postbiotics, or faecal microbiota transplantation) improve anaemia recovery or reduce invasive bacterial infection?
- Which biomarkers best identify gut barrier dysfunction, microbial translocation, immune dysregulation, and risk of invasive bacterial infection in severe anaemia?
- Can integrated biomarker panels improve risk stratification and guide personalised management?

## Figures and Tables

**Table 1:** Summary of studies examining the association between anaemia (and its major aetiologies in sub-Saharan Africa) and the gut microbiome in humans.

Ref	Country	Study Design	Study Period	Age Range	Sample Size	Anaemia Definition	Sequencing Method (16S Region)	Gut Taxa Enriched	Gut Taxa Reduced	Study Limitations
Anaemia ^1^										
[65]	Cambodia	RCT	Nov 2012–Jul 2013	6–14 years	76	NS	Illumina MiSeq (V3–V5)	Genus: *Anaerostipes, Prevotella, Ruminococcus torques* group	Genus: Limosilactobacillus, Erysipelatoclostridiaceae and Klebsiella.	(1) Functional capacity inferred, not measured. (2) Limited taxonomic resolution.
[64]	Peru	Case–control	NS	<10 years	18	Hb < 11 g/dL	Illumina (V3– V4)	Order: Clostridiales	Clostridia class and Peptostreptococcales-Tissierellales	(1) Small sample size. (2) Only taxonomical profiling. (3) Cross-sectional design.
[67]	USA	Case–control	Aug 2016–Aug 2021	25–29 weeks (gestational age)	38	Severe anaemia: Hct ≤ 25% at 14 days	Illumina NextSeq 2000 (NS)	No effect	No effect	(1) Small sample size. (2) Prematurity introduces substantial confounding. (3) No direct assessment of barrier function
[66]	USA	Cohort	May 2012–Dec 2013	0–8 weeks	80	Anaemia: Hct < 30% Severe anaemia: Hct ≤ 25%	Illumina Miseq (V4–V5)	Phylum (Anaemia): Proteobacteria [*Klebsiella*] (4–8 weeks) Phylum (Severe anaemia): Proteobacteria	Anaemia: Firmicutes (Baseline, 2–4 weeks; 4–8 weeks); Clostridium (baseline)	(1) Prematurity introduces substantial confounding. (2) Only taxonomical profiling.
Iron deficiency anaemia ^1^										
[73]	China	Case–control	May 2019–Jun 2020	Pregnant women	30	Hb < 11 g/dL	Illumina HiSeq 2500 (NS)	Genus: *Blautia, Eubacterium, Streptococcus, Olsenella, Massilioclostridium, Bifidobacterium, Actinomyces, Parvimonas, Eggerthia, Rothia, Gemella, Atopobium, Staphylococcus, Massiliomicrobiota*	Genus: *Bacteroides*	(1) Small sample size. (2) Cross-sectional design. (3) Confounding from pregnancy.
[71]	India	Case–control	NS	18–25 years	34	Hb ≤ 100 g/L	NS	*Species: Lactobacillus acidophilus*	Genus: *Bifidobacterium;* Species: *Bacteroides– Prevotella group*, *E. rectale* and *C. leptum*	(1) Small sample size. (2) Cross-sectional design. (3) Targeted microbiological assays. (4) Only taxonomical profiling.
[74]	Korea	Case–control	NS	20–50 years	31	NS	iSeq 100 platform (V4)	Genus: *Veillonella*	Genus: *Faecalibacterium, Collinsella*	(1) Small sample size. (2) Cross-sectional design. (3) Only taxonomical profiling.
[72]	Lithuania	Case–control	NS	6–34 months	20	Hb < 110 g/L	Illumina MiSeq (V3–V4)	Family: Enterobacteriaceae and Veillonellaceae	Family: Coriobacteriaceae, Bifidobacteriaceae/Enterobacteriaceae ratio	(1) Small sample size. (2) Cross-sectional design. (3) Only taxonomical profiling.
[63]	Peru	Case–control	Aug–Nov 2014	12–13 months	68	Hb < 110 g/L	Illumina MiSeq (V4)	NS	Genus (males): Coprococcus, Dorea, Roseburia, Desulfovibrio Genus (Females): Butyricicoccus	(1) Limited longitudinal assessment.
[75]	South Africa	Case–control	NS	8–13 years	166	Hb < 115 g/L	Illumina MiSeq (V4)	NS	Genus: *Anaerostipes, Anaerotruncus, Fusicatenibacter*	(1) Confounding by HIV infection/treatment (2) Microbiome function not measured
Severe malarial anaemia										
[81]	Uganda	Cohort	NS	0.5–4 years	75	Severe anaemia: Hb ≤ 5 g/dL	MVRSION (All 9 hypervariable regions)	Species: *Escherichia coli*, *Parabacteroides distasonis*, *Bacteroides caccae*, and *Klebsiella pneumoniae*	NS	(1) Only taxonomical profiling.
Sickle cell disease ^1^										
[77]	Angola	Case–control	NS	3–14 years	72	NS	NextSeq550 (NS)	Genus: Actinobacteria, Clostridium cluster XI	Genus: Aestuariispira, Campylobacter, Helicobacter, Polaribacter, and Anaerorhabdus	(1) Cross-sectional design. (2) Only taxonomical profiling. (3) No anaemia definition or classification
[79]	USA	Case–control	Jun–Sep 2016	22–58 years	28	NS	Illumina HiSeq (V3–V4)	Family: Acetobacteraceae, Acidaminococcaceae, Actinomycetaceae, Bacteroidaceae, Bifidobacteriaceae, Fusobacteriaceae, Peptostreptococcaceae, and Veillonellaceae	Family: Pasteurellaceae, Bacillaceae, Desulfovibrionaceae, Christensenellaceae, Victivallaceae, Methanobacteriaceae, Oxalobacteriaceae, Kopriimonadaceae, Verrucomicrobiaceae, Prevotellaceae	(1) Small sample size. (2) Cross-sectional design. (3) Only taxonomical profiling. (4) No anaemia definition or classification
[80]	USA	Case–control	Nov 2021–Jun 2022	4–18 years	41	NS	NS	Species: *Veillonella dispar*, *Eubacterium dolichum*, *Eggerthella lenta*, *Streptococcus anginosus* (Before FDR)	Species: *Dorea formicigenerans* (Before FDR adjustment)	(1) Small sample size. (2) Cross-sectional design. (3) Only taxonomical profiling. (4) No anaemia definition or classification
[76]	USA	Case–control	NS	>8 years	50	NS	NS	Genus: *Escherichia–Shigella*	Genus: Pseudobutyrivibrio, Faecalibacterium, Subdoligranulum, Prevotella 9, Alistipes	(1) Small sample size. (2) Cross-sectional design. (3) Only taxonomical profiling. (4) No anaemia definition or classification

^1^ Includes all anaemia severities. Abbreviations: NS, not stated; Hb, haemoglobin; MVRSION, multiple 16S variable region species level identification; Hct, haematocrit; RCT, randomised controlled trial; FDR, false discovery rate; USA, United States of America.

**Table 2:** Disease-specific biological drivers and shared gut microbiome alterations in major causes of severe anaemia.

Severe Anaemia Aetiology	Predominant Biological Driver(s)	Gut Changes ^1^	Evidence Strength	Key Uncertainty
All-cause severe anaemia	Tissue hypoxia, immune dysfunction, disrupted iron homeostasis	↓ microbial diversity, ↑ pathobionts, ↓ SCFA-producing commensals, barrier dysfunction, intestinal inflammation	Limited (human observational + experimental studies)	Whether severe anaemia independently drives dysbiosis
Iron deficiency	Reduced systemic and luminal iron availability	↓ microbial diversity, ↓ SCFA producers, ↑ pathobionts, altered microbial metabolism	Moderate (human observational + experimental studies)	Relative contribution of iron deficiency vs. anaemia
Severe malarial anaemia	Haemolysis, parasite-induced inflammation	↑ pathobionts, barrier dysfunction, endotoxaemia	Limited (human observational studies)	What are the effects of malaria vs. severe anaemia
Sickle cell disease	Chronic haemolysis, vaso-occlusion, intestinal hypoxia	Dysbiosis, barrier dysfunction, inflammation	Moderate (human observational + experimental studies)	Effects of sickle cell disease vs. chronic anaemia
Malnutrition-associated anaemia	Nutrient deficiency, oxidative stress-mediated haemolysis, EED	Immature microbiome, barrier dysfunction, dysbiosis	Limited (human observational studies)	Independent role of anaemia

^1^ Upwards arrows (↑) indicate increased activity; downward arrows (↓) indicate reduced or suppressed activity.

**Table 3:** Studies examining the effects of iron supplementation and other iron interventions on the gut microbiome in children.

Ref	Country	Study Period	Age Range	Sample Size	Treatment (*n*)	Duration	Controls (*n*)	Sequencing Method (16S Region)	Effect of Iron on Gut Microbiota	Other Effect on Gut
[167]	Bangladeshi	Sep 2018–Feb 2019	8 months	1093	(1) 12.5 mg ferrous sulphate (*n* = 308) (2) 12.5 mg ferrous fumarate (*n* = 307)	3 months	Placebo (*n* = 308)	Illumina MiSeq, NovaSeq (V4)	No effect (reduced commensals in unadjusted model)	No effect on diarrhoea incidence
[97]	Cote d’Ivoire	Nov 2006–Jun 2007	6–14 years	139	(2) iron fortified biscuits, 20 mg Fe/d (*n =* 70)	6 months	Unfortified biscuits, (*n =* 69)	PCR (V2–V3)	Increased Enterobacteria and decreased Lactobacilli	Elevated mean faecal calprotectin or GI illness
[163]	Kenya	Apr 2011–Jan 2013	6 months	33	MNP + 12.5 mg Fe (*n* = 13)	3 months	(1) MNP without Fe (*n* = 13) (2) Placebo (*n* = 7)	Illumina Miseq (V4)	Decreased Escherichia/Shigella in control groups, but not MNP + Fe	No group differences in faecal calprotectin
[165]	Kenya	Oct 2014–Dec 2015	6.5–9.5 months	145	(1) 2.5 mg iron as NaFeEDTA (*n* = 49) (2) GOS + 2.5 mg as ferrous fumarate (*n =* 48)	4 months	MNP without iron (*n* = 48)	Illumina MiSeq (V3–V4)	Addition of GOS mitigated most of the adverse effects of iron on the gut microbiome.	No group differences in faecal calprotectin or diarrhoea treatment
[96]	Kenya	Mar 2010–Sep 2011	5.5 months	101	(1) MNP with 2.5 mg Fe as NaFeEDTA (*n* = 28) (2) MNP with 12.5 mg Fe as ferrous fumarate (*n* = 21)	4 months	(1) MNP without iron (*n* = 26) (2) MNP without iron (*n* = 26)	Titanium pyrosequencing chemistry (V3–V6)	Increase in the sum of pathogenic *E. coli*	Greater incidence of treated diarrhoea episodes, and elevated faecal calprotectin in MNP + 12.5 mg Fe group
[168]	South Africa	Feb–Nov 2010	6–11 years	73	50 mg Fe as FeSO_4_ (*n* = 22)	38 weeks	(1) Placebo (*n* = 27) (2) Iron sufficient group (*n* = 24)	PCR (NS)	No effect	No group differences in faecal calprotectin or diarrhoea illness
[166]	Sweden	NS	6 months	72	(1) Low iron: 1.2 mg Fe/day (2) High iron: 6.6 mg Fe/day		Iron drops (no–added–iron formula 6.6 mg Fe/day)	Illumina MiSeq (V3–V4)	High iron group: Increased Streptococcus, reduced Clostridium and Bacteroides	No group differences in faecal calprotectin

Abbreviations: NS, not stated; GOS, galacto-oligosaccharide; MNP, micronutrient powder; Fe, iron; Zn, Zinc; PCR, polymerase chain reaction; GI, gastrointestinal; USA, United States of America.

## Data Availability

No new data were created or analysed in this study. Data sharing is not applicable to this article.

## Abbreviations

The following abbreviations are used in this manuscript:

DMT1	Divalent metal transporter 1
EED	Environmental enteric dysfunction
FDR	False discovery rate
FPN	Ferroportin
FR	Ferric reductase
Hb	Haemoglobin
Hct	Haematocrit
Heph	Hephaestin
IDA	Iron deficiency anaemia
IgA	Immunoglobulin A
LPS	Lipopolysaccharide
MVRSION	Multiple 16S variable region species level identification
NS	Not stated
NTBI	Non-transferrin-bound iron
NTS	Non-typhoidal Salmonella
RBC	Red blood cell
RCT	Randomised controlled trial
SCA	Sickle cell anaemia
SCFA	Short-chain fatty acid
SMA	Severe malarial anaemia
USA	United States of America
